# Durability and Effectiveness of Maraviroc-Containing Regimens in HIV-1-Infected Individuals with Virological Failure in Routine Clinical Practice

**DOI:** 10.1371/journal.pone.0144746

**Published:** 2015-12-29

**Authors:** Valérie Potard, Jacques Reynes, Tristan Ferry, Céline Aubin, Laurent Finkielsztejn, Yazdan Yazdanpanah, Dominique Costagliola, FHDH ANRS CO4

**Affiliations:** 1 Sorbonne Universités, UPMC Univ Paris 06, UMR_S 1136, Paris, France; 2 INSERM, UMR_S 1136, Institut Pierre Louis d'Epidémiologie et de Santé Publique, Paris, France; 3 Inserm Transfert, Paris, France; 4 Université Montpellier, Montpellier, France; 5 IRD, UMI233 TransVIHMI Montpellier, France; 6 Département de Maladies Infectieuses et Tropicales, CHU Montpellier, France; 7 Services de Maladies Infectieuses et Tropicales, Hospices Civils de Lyon; Université Claude Bernard Lyon1; CIRI, International Center for Infectiology Research, INSERM U1111, Lyon, France; 8 Laboratoire GSK, Marly-Le-Roi, France; 9 Laboratoire ViiVHealthcare, Marly-Le-Roi, France; 10 Université Paris Diderot 7, Paris, France; 11 INSERM, UMR_S 1137, ATIP-Avenir Inserm: "Modélisation, Aide à la Décision, et Coût-Efficacité en Maladies Infectieuses”, Paris, France; 12 Service des Maladies Infectieuses et Tropicales, Hôpital Bichat, France; University of British Columbia, CANADA

## Abstract

**Introduction:**

Limited data are available on the durability and effectiveness of maraviroc in routine clinical practice. We assessed the durability of maraviroc-containing regimens during a 30-month period, as well as their immunovirological and clinical efficacy, according to viral tropism in treatment-experienced individuals with viral load (VL) >50 copies/ml in the French Hospital Database on HIV.

**Methods:**

Virological success was defined as VL<50 copies/ml, immunological success as a confirmed increase of at least 100 CD4 cells/mm^3^ measured twice at least one month apart, and clinical failure as hospitalization for a non-AIDS event, an AIDS event, or death. Multivariable Cox regression models adjusted for potential confounders were used to assess the influence of viral tropism on durability, the immunovirological responses, and clinical outcome.

**Results:**

356 individuals started maraviroc with VL>50 copies/ml of whom 223 harbored R5 viruses, 44 non-R5 viruses and 89 viruses of unknown tropism. Individuals with non-R5 viruses were more likely than individuals with R5 viruses to discontinue maraviroc (75% vs 34%, p<0.0001). At 30 months, the estimated rates of virological and immunological success were respectively 89% and 51% in individuals with R5 viruses and 48% and 23% in individuals with non-R5 viruses. In multivariable analysis, non-R5 viruses were associated with a lower likelihood of both virological success (hazard ratio (HR): 0.42; 95% confidence interval (CI), 0.25–0.70) and immunological success (HR: 0.37; 95% CI, 0.18–0.77). No difference in clinical outcome was found between individuals with R5 and non-R5 viruses. The effectiveness of maraviroc-containing regimens in individuals with unknown viral tropism was not significantly different from that in individuals with R5 viruses. A limitation of the study is the absence of genotypic susceptibility score.

**Conclusion:**

In this observational study, maraviroc-containing regimens yielded high rates of viral suppression and immunological responses in individuals with R5 viruses in whom prior regimens had failed.

## Introduction

The primary aims of antiretroviral therapy (ART) for HIV infection are to reduce morbidity and prolong life by reducing viral load and restoring the CD4+ T cell count with minimal toxicity/adverse events. Combination antiretroviral therapy (cART) suppresses viral load and delays disease progression, but efficacy can be undermined by the occurrence of drug resistance [1]. New drugs with different mechanisms of action provide options for individuals with drug-resistant HIV [2,3]. Maraviroc is a first-in-class selective antagonist of the chemokine coreceptor type-5 (CCR5). Maraviroc changes the CCR5 receptor conformation and thereby prevents HIV envelope protein binding and virus entry. Maraviroc showed antiretroviral activity in early phase 2a studies in HIV-infected individuals who were treatment-naive or had been off treatment for at least 8 weeks, and who harbored CCR5-tropic (R5) viruses [4]. Maraviroc approval for treatment-experienced individuals with R5 viruses was based on the MOTIVATE I and II placebo-controlled trials which showed virologic efficacy at 48 weeks [5].

As maraviroc efficacy is dependent on R5 receptor usage for cell entry, it is necessary to determine the tropism of the individual’s virus. In France, genotypic tropism assays based on the V3 region of the HIV envelope protein are reimbursed by social security.

CCR5 antagonists are also being developed as immunomodulatory and antiinflammatory agents. Clinical studies [6–12] have shown a larger increase in the CD4+ T cell count in individuals treated with CCR5 antagonists as compared to other antiretrovirals but this difference is not clearly explained by the virological response. It is important to assess how maraviroc is used in routine care settings, and to evaluate its biological and clinical impact, as a complement to clinical trials [13].

The purpose of this study was to describe the routine use of maraviroc in treatment- experienced HIV-infected individuals with treatment failure, and to assess its effectiveness in terms of durability, virological and immunological responses, and clinical outcome according to viral tropism.

## Individuals and Methods

### Individuals and data sources

The French Hospital Database on HIV (FHDH) is a hospital-based multicentre open cohort in which inclusions have been ongoing since 1989 [14]. It includes data from 70 general or university hospitals distributed throughout France. Individuals are eligible if they have documented HIV-1 or HIV-2 infection and give their written informed consent to participate. Data are collected prospectively by trained research assistants using standardized forms and include demographic characteristics, biological markers such as the CD4 cell count and plasma HIV RNA level, the date and type of AIDS and non AIDS-defining events, antiretroviral treatments, and the date and causes of death. The FHDH project was approved by the French computer watchdog authority (CNIL) on 27 November 1991 (Journal Officiel, 17 January 1992).

### Study population

This study was restricted to antiretroviral-experienced HIV-1-infected individuals, at least 18 years of age who started maraviroc-based therapy with a viral load (VL) of >50 copies/ml between January 1^st^, 2008 and June 30^th^, 2011, at least one year before the last recorded FHDH visit in the center, with an available CD4 cell count within 6 months before maraviroc initiation and at least one CD4 cell count and one VL measurement after maraviroc initiation. HIV-1 coreceptor tropism test execution (yes/no), assay type (phenotypic/genotypic) and result were collected from the medical records. The phenotypic assay was Trofile^™^ (Monogram Biosciences). The genotypic assay was mainly geno2pheno with a false-positive rate (FPR) cut-off of 10%, which was shown in a study of the ANRS AC11 network of virology laboratories to have good predictive value as compared with the Trofile phenotypic test [15]. The genotypic test is used routinely in the French network of virology laboratories and the cost is covered by social security, while the phenotypic test is used solely as a research tool.

### Statistical analysis

The baseline for all analyses was the date of maraviroc treatment initiation. Tropism was classified in 3 categories: R5; non-R5 if X4 or dual; and unknown tropism if not tested or if the results were unavailable. Baseline characteristics were analysed according to viral tropism.

The following endpoints were studied: durability, defined in terms of maraviroc discontinuation; virological response (VL <50 copies/ml); immunological response (confirmed increase of at least 100 CD4 cells/mm^3^ measured twice at least one month apart); and clinical outcome (AIDS-defining event [16], hospitalization for a non AIDS event, or death). We took into account all hospital admissions lasting more than 24 hours for non-AIDS events, excluding pregnancy and diagnostic examinations. We used the diagnostic code reported in the medical hospital information system. For the most common events (cancer, cardiovascular disease, etc), regular audits, showed good-quality coding with validation rates over 90%.

We calculated Kaplan-Meier estimates for the rates of maraviroc discontinuation, virological response, immunological response and clinical outcome at 30 months. For virological and immunological responses, maraviroc discontinuation could be considered as a competing event (individuals who discontinue treatment are likely to be those experiencing a slower reduction in viral load or a slower increase in the CD4 cell count). Therefore, a competing-risk approach was adopted when evaluating biological outcomes. In this approach, if maraviroc was discontinued, follow-up was right-censored at the date of the individual's last visit during the 30-month follow-up period. This approach ensures that no endpoint is recorded during the period between maraviroc discontinuation and the end of the 30-month follow-up period, thus avoiding a situation in which the majority of individuals switch from maraviroc and achieve a viral load reduction on an alternative treatment. For the clinical endpoint, we used an intention-to-continue-treatment approach, ignoring treatment change in order to adopt a conservative approach as for the analyses of biological outcomes. Univariable and multivariable Cox regression models were used to assess the influence of viral tropism (R5, non-R5, unknown) on the durability of maraviroc-containing regimens, and on virological response, immunological response and clinical outcome. The following potential confounding factors were analyzed: the type of treatment change (addition of MVC / switch to MVC with no other change / switch to MVC + other new drug(s)), gender, age, sub-Saharan origin, AIDS status, HCV co-infection, the nadir CD4 T cell count (<100/mm^3^, ≥ 100/mm^3^), baseline CD4 T cell count (<200/mm^3^, ≥ 200/mm^3^), baseline VL (log_10_ transformed, as a continous variable), use of prophylaxis for opportunistic infections, number of previous antiretroviral drugs, duration of prior antiretroviral exposure, and co-prescribed NRTIs, boosted PI, RAL, and ETV at baseline. All these variables were retained for multivariable analysis. Sensitivity analyses were performed by splitting the results for individuals with “unknown tropism” into two categories: “unavailable” (no knowledge of whether or not a test was performed) and “not tested”. SAS software (v9.2; SAS Institute Inc, Cary, NC, USA) was used for all statistical analyses.

## Results

### Baseline characteristics

**Fig 1:**
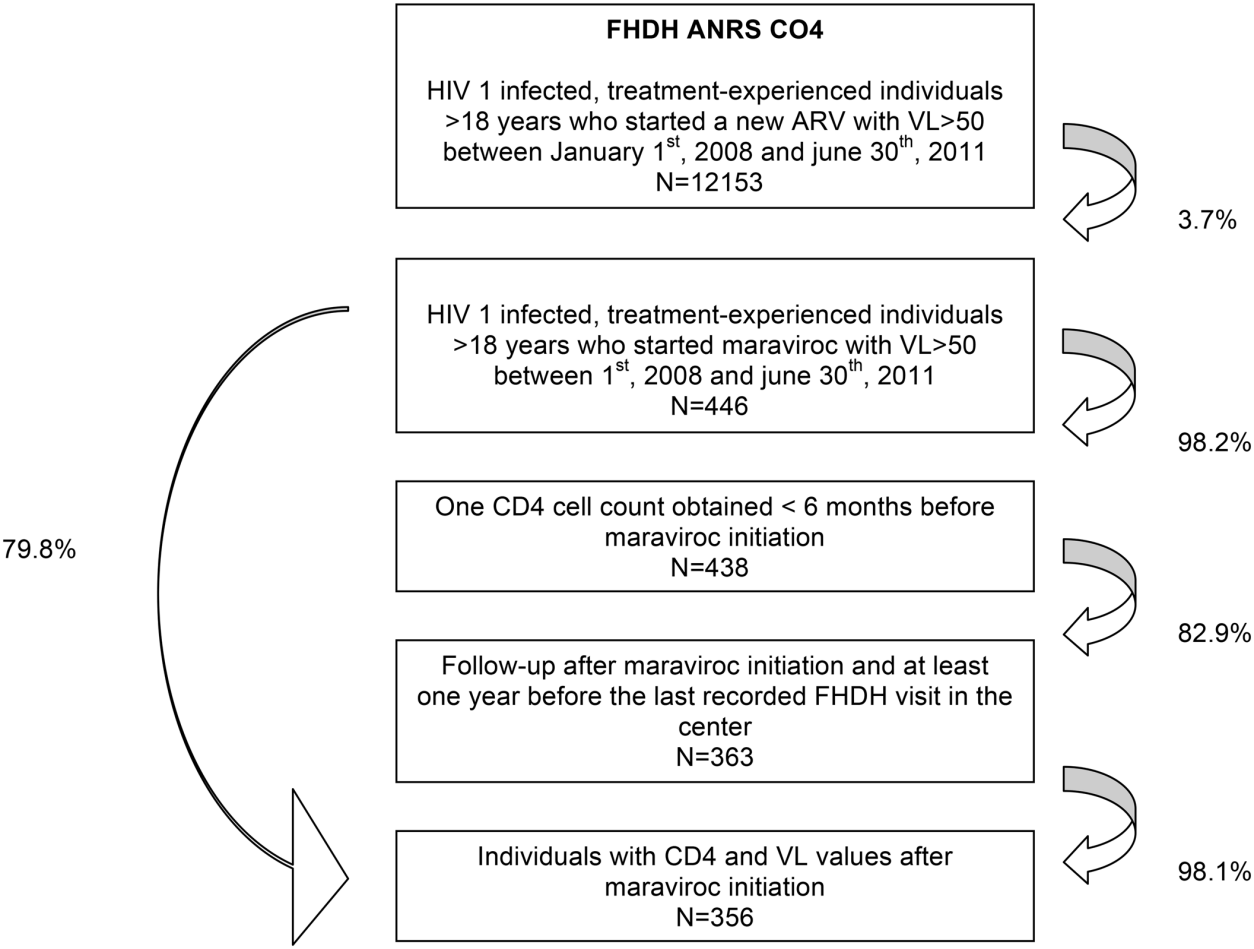
Individual selection.

The flow chart of the study is shown in Fig 1. A total of 356 individuals were included, of whom 223 (62.6%) had R5 viruses and 44 (12.4%) non-R5 viruses. Among the 89 individuals (25.0%) with unknown viral tropism, information on test performance (yes/no) was missing from the medical records in 53 cases, the test was not performed in 30 cases, and the result was indeterminate in 6 cases. Among the 267 individuals with known viral tropism, a phenotypic assay was used in 61 cases (22.9%), a genotypic assay in 201 cases (75.3%), both a phenotypic and a genotypic assay in 2 cases (0.8%) and an unknown assay in 3 cases (1.1%).

The individuals’ baseline characteristics are shown in Table 1. Individuals with non-R5 viruses had more advanced HIV infection, with lower CD4 cell counts (median 147 versus 330/mm^3^), lower nadir CD4 cell counts (median 17 versus 102/mm^3^) and more frequent AIDS status (63.6% versus 45.3%) than individuals with R5 viruses. Individuals with non-R5 viruses were more likely to be co-prescribed at least three antiretrovirals (84.1% versus 52.5%). The maraviroc-based regimens included at least one NRTI in 68.0% of cases, one PI in 64.6% (darunavir in 40.2%), etravirine in 31.2%, raltegravir in 52.0% and T20 in 8.4%. Median follow-up was 21.4 months (IQR: 14.9–30.4).

**Table 1:** Individual characteristics at maraviroc initiation according to viral tropism.

	VL>50	VL>50 and R5 viral tropism	VL>50 and non- R5 viral tropism	VL>50 and unknown viral tropism^a^	P
	n = 356	n = 223	n = 44	n = 89	
**Type of treatment change**					
Addition of maraviroc	117 (32.9%)	61 (27.4%)	21 (47.7%)	35 (39.3%)	**0.04**
Switch to maraviroc with no other change	56 (15.7%)	38 (17.0%)	7 (15.9%)	11 (12.4%)	
Switch to maraviroc + other new drug(s)	183 (51.4%)	124 (55.6%)	16 (36.4%)	43 (48.3%)	
**Gender**					
F	114 (32.0%)	71 (31.8%)	13 (29.5%)	30 (33.7%)	0.89
**Age** (years) **(**Med. [IQR])	46.5 [41.5–52.1]	47.0 [42.2–53.1]	45.6 [40.8–50.9]	44.7 [41.3–50.6]	0.22
**Transmission group**					
MSM	131 (36.8%)	83 (37.2%)	16 (36.4%)	32 (36.0%)	0.95
IDU	45 (12.6%)	30 (13.5%)	4 (9.1%)	11 (12.4%)	
Heterosexual	140 (39.3%)	88 (39.5%)	18 (40.9%)	34 (38.2%)	
Other	40 (11.2%)	22 (9.9%)	6 (13.6%)	12 (13.5%)	
**Sub-Saharan origin**					
Yes	40 (11.2%)	18 (8.1%)	8 (18.2%)	14 (15.7%)	**0.05**
**HCV co-infection**					
Yes	64 (18.0%)	41 (18.4%)	8 (18.2%)	15 (16.9%)	0.95
**AIDS status**					
Yes	177 (49.7%)	101 (45.3%)	28 (63.6%)	48 (53.9%)	**0.05**
**CD4 T-cell nadir/**mm^3^ **(**Med. [IQR])	85 [19–182]	102 [26–215]	17 [3–77]	82 [20–145]	**<0.0001**
Nadir CD4 < 100/mm^3^	194 (54.5%)	107 (48.0%)	37 (84.1%)	50 (56.2%)	**0.0001**
**CD4 T-cell count/**mm^3^ **(**Med. [IQR])	289 [151–462]	330 [180–472]	147 [71–300]	269 [150–507]	**0.0003**
CD4< 200/mm^3^	124 (34.8%)	65 (29.1%)	28 (63.6%)	31 (34.8%)	**0.0001**
**Plasma HIV-1 RNA** (log_10_ copies/ml) **(**Med. [IQR])	3.3 [2.3–4.5]	3.3 [2.3–4.4]	3.3 [2.2–4.8]	3.6 [2.5–4.6]	0.45
VL≥100000 copies/ml	50 (14.0%)	29 (13.0%)	6 (13.6%)	15 (16.9%)	0.67
**Time since HIV diagnosis** (years) **(**Med. [IQR])	17 [13–21]	17 [13–20]	17 [11–20]	18 [13–21]	0.73
**OI prophylaxis**					
Yes	86 (24.2%)	46 (20.6%)	18 (40.9%)	22 (24.7%)	**0.02**
**First ART**					
Mono/bitherapy	201 (56.5%)	122 (54.7%)	22 (50.0%)	57 (64.0%)	0.21
cART	155 (43.5%)	101 (45.3%)	22 (50.0%)	32 (36.0%)	
**Number of past ARV drugs** ^b^ **(**Med. [IQR])	11 [7–15]	11 [7–15]	14 [7–18]	12 [8–15]	0.06
< = 10	158 (44.4%)	109 (48.9%)	16 (36.4%)	33 (37.1%)	0.09
> 10	198 (55.6%)	114 (51.1%)	28 (63.6%)	56 (62.9%)	
**Duration of prior antiretroviral exposure** (years) **(**Med. [IQR])	13 [8–15]	12 [8–15]	12 [6–14]	13 [8–15]	0.15
**Number of other prescribed drugs** at baseline ^b^	3 [2–4]	3 [2–3]	3 [3–4]	3 [2–4]	**<0.0001**
1	27 (7.6%)	18 (8.1%)	0 (0%)	9 (10.1%)	**0.0002**
2	113 (31.7%)	88 (39.5%)	7 (15.9%)	18 (20.2%)	
3	120 (33.7%)	68 (30.5%)	16 (36.4%)	36 (40.4%)	
>3	96 (27.0%)	49 (22.0%)	21 (47.7%)	26 (29.2%)	
**NRTI**	242 (68.0%)	142 (63.7%)	37 (84.1%)	63 (70.8%)	**0.02**
* 1*	*54 (15*.*2%)*	*33 (14*.*8%)*	*7 (15*.*9%)*	*14 (15*.*7%)*	
* 2*	*173 (48*.*6%)*	*102 (45*.*7%)*	*26 (59*.*1%)*	*45 (50*.*6%)*	
* >2*	*15 (4*.*2%)*	*7 (3*.*1%)*	*4 (9*.*1%)*	*4 (4*.*6%)*	
**Boosted PI**	232 (65.2%)	141 (63.2%)	30 (68.2%)	61 (68.5%)	0.51
* DRV*	*144 (40*.*4%)*	*90 (40*.*4%)*	*17 (38*.*6%)*	*37 (41*.*6%)*	
* ATV*	*18 (5*.*1%)*	*12 (5*.*4%)*	*2 (4*.*5%)*	*4 (4*.*5%)*	
* LPV*	*39 (11*.*0%)*	*18 (8*.*1%)*	*8 (18*.*2%)*	*13 (14*.*6%)*	
* TPV*	*21 (5*.*9%)*	*14 (6*.*3%)*	*3 (6*.*8%)*	*4 (4*.*5%)*	
* Other (FPV*, *IDV*, *SQV)*	*10 (2*.*8%)*	*7 (3*.*1%)*	*0 (0%)*	*3 (3*.*4%)*	
**Raltegravir**	185 (52.0%)	118 (52.9%)	20 (45.5%)	47 (52.8%)	0.65
**Etravirine**	111 (31.2%)	65 (29.1%)	26 (59.1%)	20 (22.5%)	**0.0001**
**T20**	30 (8.4%)	13 (5.8%)	6 (13.6%)	11 (12.4%)	0.07

P values for comparison across the three groups (R5, non-R5, unknown viral tropism), chi-square test for categorical variables and Kruskall Wallis test for continuous variables.

^a^ 53 unavailable data (59.6%), 30 not tested (33.7%) and 6 indeterminate results (6.7%)

^b^ Not counting boosted ritonavir

### Durability of maraviroc-containing regimens

**Fig 2:**
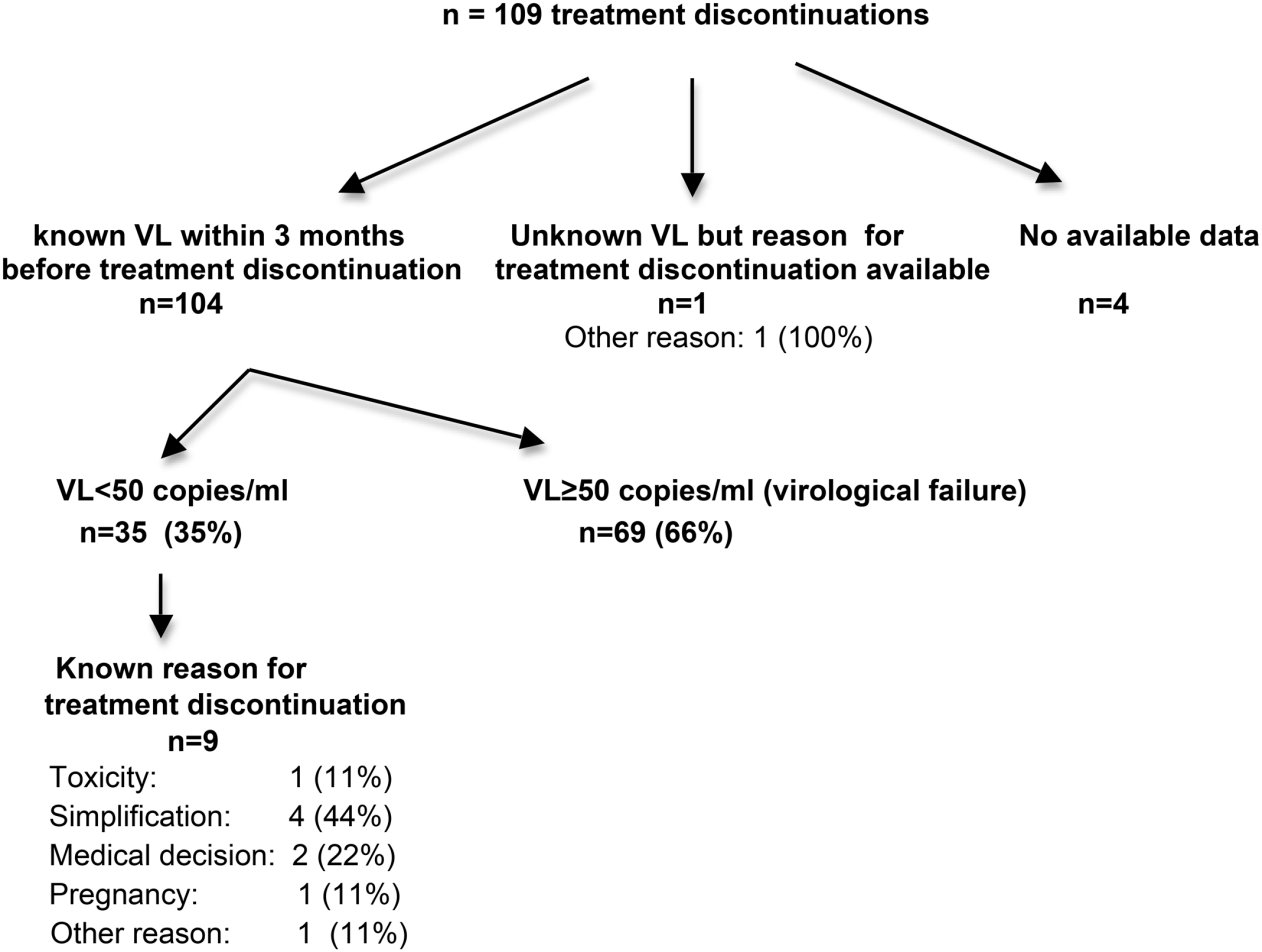
Discontinuations.

Among the 356 individuals who started maraviroc therapy, 109 individuals discontinued treatment. The estimated rate of maraviroc discontinuation at month 30 was 41%. The flow chart in Fig 2 shows treatment discontinuations according to viral load within 3 months before discontinuation and the reasons for discontinuation, when available. Among the 104 individuals who discontinued maraviroc and who had an available VL value obtained within the previous 3 months, 35 individuals (35%) discontinued maraviroc after achieving VL<50 copies/ml.

**Fig 3:**
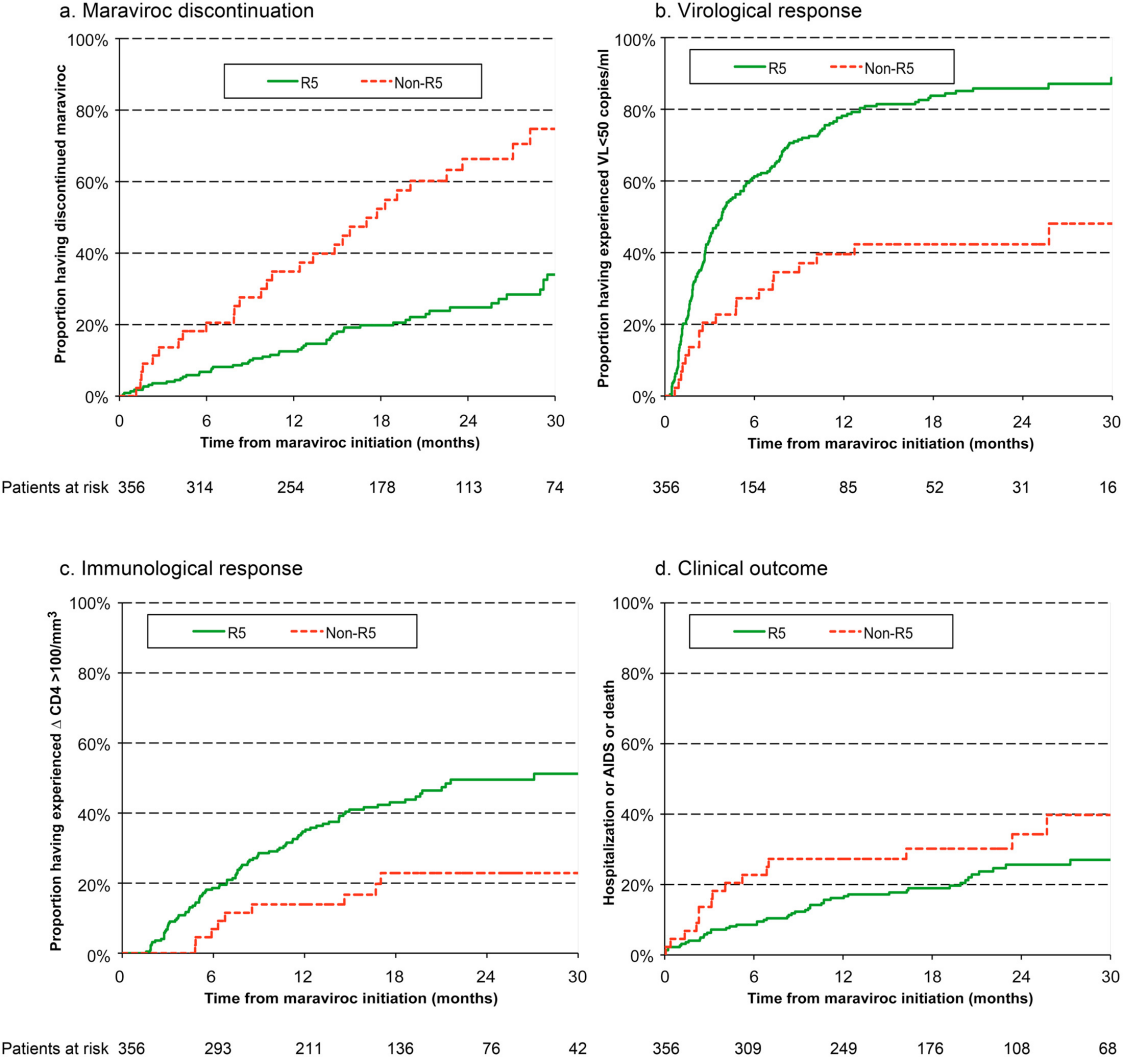
Kaplan-Meier plots showing, according to R5 or non-R5 viral tropism, the times (a) to maraviroc discontinuation, (b) to a virological response VL<50 copies/ml, (c) to a sustained gain of at least 100 CD4 cells/mm^3^, (d) to hospitalization for a non AIDS event, an AIDS event or death.

**Fig 4:**
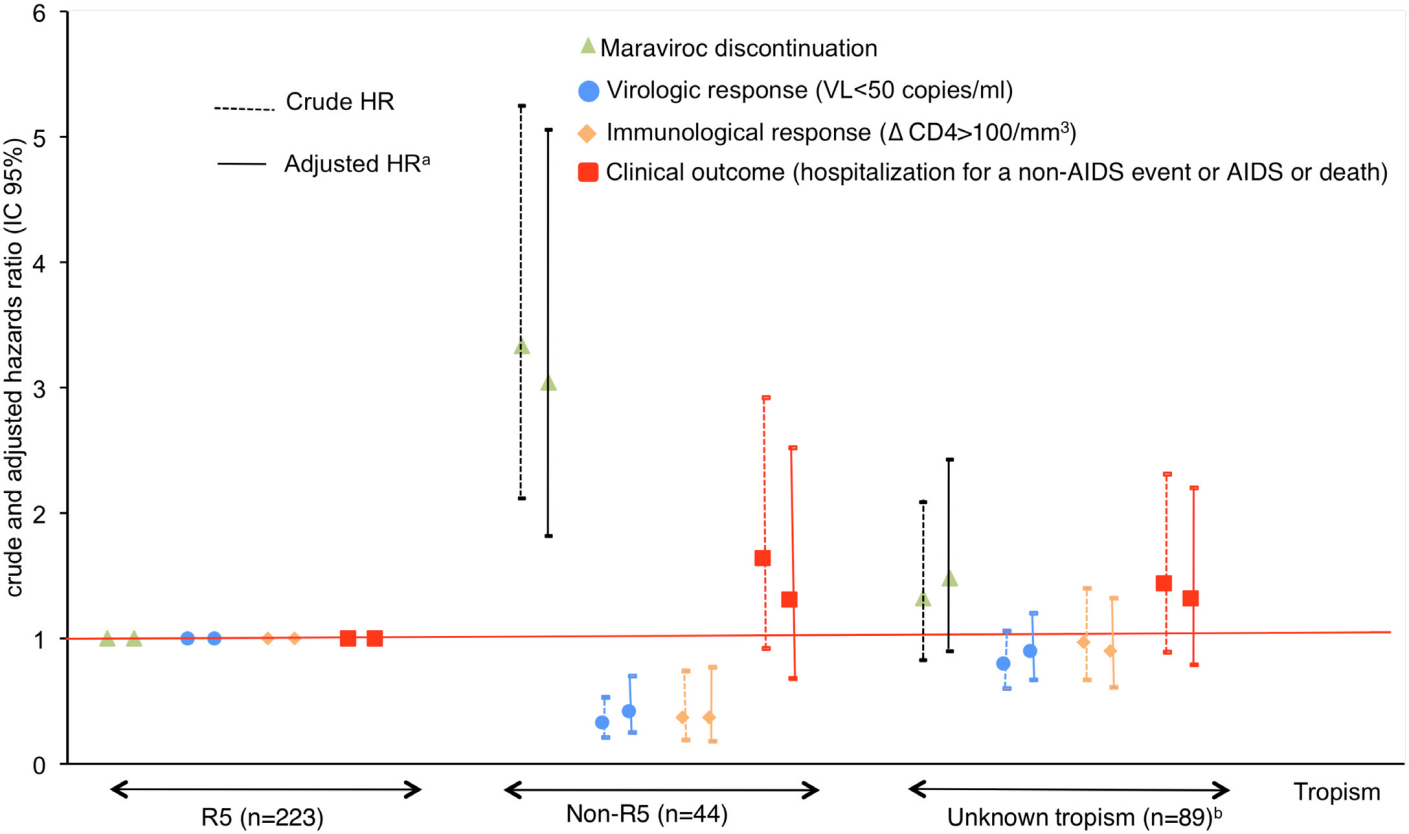
Durability, immunovirological response and clinical outcomes of maraviroc-based regimens up to month 30 according to viral tropism. ^a^ Adjusted on type of treatment change, gender, age, Sub-Saharan origin, HCV co-infection, AIDS status, CD4 nadir, CD4, plasma HIV-1 RNA, OI prophylaxis, number of past ARV drugs, duration of prior ARV exposure, NRTI, boosted PI, raltegravir, etravirine at baseline. ^b^ 53 unknown viral tropism because unavailable data; 30 because viral tropism was not tested; 6 because the result of test was indeterminate.

The estimated rates of maraviroc discontinuation before month 30 were 34% among individuals with R5 viruses and 75% among those with non-R5 viruses (Fig 3a). Individuals with non-R5 viruses were significantly more likely to discontinue maraviroc than individuals with R5 viruses (adjusted HR = 3.04; 95% confidence interval (CI), 1.82–5.06) (Fig 4). The durability of maraviroc-containing regimens was similar in individuals with unknown viral tropism and individuals with R5 viruses. There was no difference in durability between individuals with unavailable tropism data and those not tested (supplementary Figure A in S1 File).

### Virological response

Viral load fell below 50 copies/ml in 268 individuals. The estimated rate of virological success at month 30 was 81% overall, with a clear difference between individuals harboring R5 and non-R5 viruses (89% versus 48%, adjusted HR 0.42 (95% CI, 0.25–0.70)) (Figs 3b and 4). The rate of virological success was not significantly different between individuals with unknown viral tropism and those with R5 viruses. There was no difference between individuals with unavailable tropism data and those not tested (supplementary Figure B in S1 File).

### Immunological response

The CD4 cell count rose by at least 100/mm^3^ in 145 individuals. The estimated rate of immunological success at month 30 was 48% overall, with a clear difference between individuals harboring R5 and non-R5 viruses (51% versus 23%, adjusted HR 0.37 (95% CI, 0.18–0.77)) (Figs 3c and 4). The rate of immunological success was not significantly different between individuals with unknown tropism and those with R5 viruses. There was no difference between individuals with unavailable tropism data and those not tested (Figure C in S1 File). The median increase in the CD4 cell count from baseline to the last measurement during the 30 month follow-up period was 98/mm^3^ [5–233] in individuals with R5 viruses, 16/mm^3^ [-61–113] in individuals with non-R5 viruses and 87/mm^3^ [10–220] in individuals with unknown viral tropism.

### Clinical outcome

During the 30-month follow-up period, 90 individuals experienced clinical failure consisting of at least one hospitalization for a non-AIDS event (n = 44), an AIDS event (n = 41) or death (n = 5). The non-AIDS events necessitating hospitalization were: infections (n = 18), malignancies (n = 4), chronic viral hepatitis (n = 3), gastrointestinal bleeding (n = 2), substance abuse, psychiatric disease, myocardial infarction or other ischemic heart disease, respiratory disease, gastrointestinal disorders (1 case each), other causes (n = 9), and unclassifiable causes (n = 3). The following AIDS events occurred: esophageal candidiasis (n = 7), encephalopathy (n = 7), cytomegalovirus disease (n = 6), Kaposi’s sarcoma (n = 4), Burkitt’s lymphona (= 4), cerebral toxoplasmosis (n = 3), extrapulmonary cryptococcosis, herpes simplex, progressive multifocal leukoencephalopathy (n = 2 each), cervical cancer, Pneumocystis jirovecii pneumonia, recurrent pneumonia, and extrapulmonary mycobacterial infection (n = 1 each). The estimated rate of clinical failure at month 30 was 30% overall, with no difference between individuals with R5 viruses and those with non-R5 viruses (27% versus 40%, adjusted HR 1.31 (95% CI, 0.68–2.52)) (Figs 3d and 4).

## Discussion

In this observational study of antiretroviral treatment-experienced individuals with VL >50 copies/ml, we evaluated the effectiveness of maraviroc-based regimens in routine clinical practice. Maraviroc was used mainly in individuals with R5 viruses. Maraviroc discontinuation was more frequent among individuals with non-R5 viruses than among those with R5 viruses. Maraviroc is sometimes prescribed within salvage regimens for individuals with non-R5 viruses and is quickly withdrawn if immune recovery is not achieved. At maraviroc initiation, individuals with non-R5 viruses had more advanced HIV infection including lower CD4 T cell counts, and most of them were prescribed four-drug regimens. The rate of maraviroc discontinuation among individuals with R5 viruses (13% at 12 months) was lower in this observational study than in the Motivate trials (37% at week 48) [5,17], possibly because individuals in the Motivate trials had viral loads above 5000 copies/ml at baseline, compared to >50 copies/ml in our study. The most common reason for maraviroc discontinuation in the Motivate trials was a lack of efficacy as judged by the patients’ physicians (61.6%), a rate similar to that found here (66.3%).

As expected, individuals with R5 viral tropism had better virological and immunological responses than individuals with non-R5 tropism. No influence of R5 status on virological and immunological responses was found in an Italian study [18], but it included only 15 individuals with non-R5 viruses. In a placebo-controlled trial of maraviroc in treatment-experienced individuals with non-R5 viruses, the reduction in viral load was similar in the maraviroc and placebo arms at week 24 [7]. In a Spanish study, a higher X4-tropic viruses level was associated with lower viral load reduction after short-term maraviroc exposure [19].

Here, among individuals harboring R5 viruses, the rate of VL suppression was 41% at 3 months and 89% at 30 months. In a recent French study of routine clinical practice, VL was <50 copies/ml at 3 months in 54% of 104 treatment-experienced patients starting maraviroc-based therapy [20]. This rate is slightly higher than that found here, but it should be noted that the baseline CD4 cell count, shown in a post-hoc analysis of the Motivate study to be a strong predictor of the virologic response [21], was lower in our study. In contrast, our virological success rate at month 30 is higher than that obtained in the Motivate trials [5,17], in which VL suppression was obtained at week 96 in 41% of individuals. Our higher rate of VL suppression may be explained by higher CD4 cell counts at maraviroc initiation and by co-administration of potent new drugs such as etravirine and raltegravir, which were not permitted as part of the optimized background therapy in the Motivate trials. The additional benefit of adding a potent new drug to the background regimen at maraviroc initiation has been shown in analyses of the virologic response according to the first use of selected background drugs [22].

At month 30, the median increase in the CD4 T cell count from baseline was +98 cells/mm^3^ in individuals harboring R5 viruses. This is similar to the increase observed at week 96 in the Motivate trials, where the median change was +113 cells/mm^3^ in individuals receiving maraviroc twice a day [17]. The median increase in the CD4 T cell count was also similar in a German study of routine clinical practice [23], with a gain of +96 cells/mm^3^ after 6 months. In an Italian study of maraviroc, raltegravir and etravirine combination therapy [8], the median increase was larger (+211 cells/mm^3^ at week 96), possibly because VL at maraviroc initiation was higher (4.2 log_10_ copies/ml) than in our study (3.3 log_10_ copies/ml) and in a German study (2.9 log_10_ copies/ml) [23]. Indeed, individuals with higher VL at treatment initiation tend to have a more rapid immunological response with a sharper slope, probably owing to CD4 T cell redistribution [24].

Regarding clinical outcome, we found no significant difference in the combined rate of hospitalization for non-AIDS events, AIDS events and death between individuals harboring R5 viruses and those with non-R5 viruses. This is surprising because the immunovirological success rate was higher in individuals harboring R5 viruses. It might be explained by rapid maraviroc discontinuation and a switch to more appropriate regimens in individuals with non-R5 viruses. Also, follow-up may have been too short or the statistical power too low to show a significant difference in clinical outcome.

The main strength of our study is its large size and routine clinical setting, providing additional evaluation of the use of maraviroc in combination with other recent drugs in treatment-experienced patients. In this observational setting, we were unable to adjust the results for the genotypic susceptibility score, or adherence. However, as we adjusted for new drug and type of treatment change, we feel our results are nevertheless robust. Another limitation is that viral tropism was unknown in 25% of cases. However, responses were similar in individuals with unrecorded tropism test performance and those known to harbor R5 viruses suggesting that most of the 15% of individuals with unknown tropism test status had in fact been shown to harbor R5 viruses, without this information being noted in the medical records. Of course, the viral tropism should always be known to allow the maraviroc prescription and the result reported in the medical records.

In conclusion, this study shows that, among HIV-infected individuals in treatment failure, maraviroc-containing regimens are generally used in those with R5 viruses, in whom they are effective in terms of durability, viral suppression, immunological recovery, and clinical responses.

## Supporting Information

S1 File Kaplan-Meier plots showing, according to viral tropism, the times (a) to maraviroc discontinuation, (b) to a virological response VL<50 copies/ml, (c) to a sustained gain of at least 100 CD4 cells/mm3 (d) to hospitalization for a non AIDS event, an AIDS event or death. (TIF)

S1 Text Clinical Epidemiology Group of the FHDH-ANRS CO4 cohort. (PDF)
