# Supplementary figures and images for: Durability and Effectiveness of Maraviroc-Containing Regimens in HIV-1-Infected Individuals with Virological Failure in Routine Clinical Practice
Source: PLoS One. 2015 Dec 29;10(12):e0144746. doi: 10.1371/journal.pone.0144746 (PMC4695083; doi:10.1371/journal.pone.0144746)

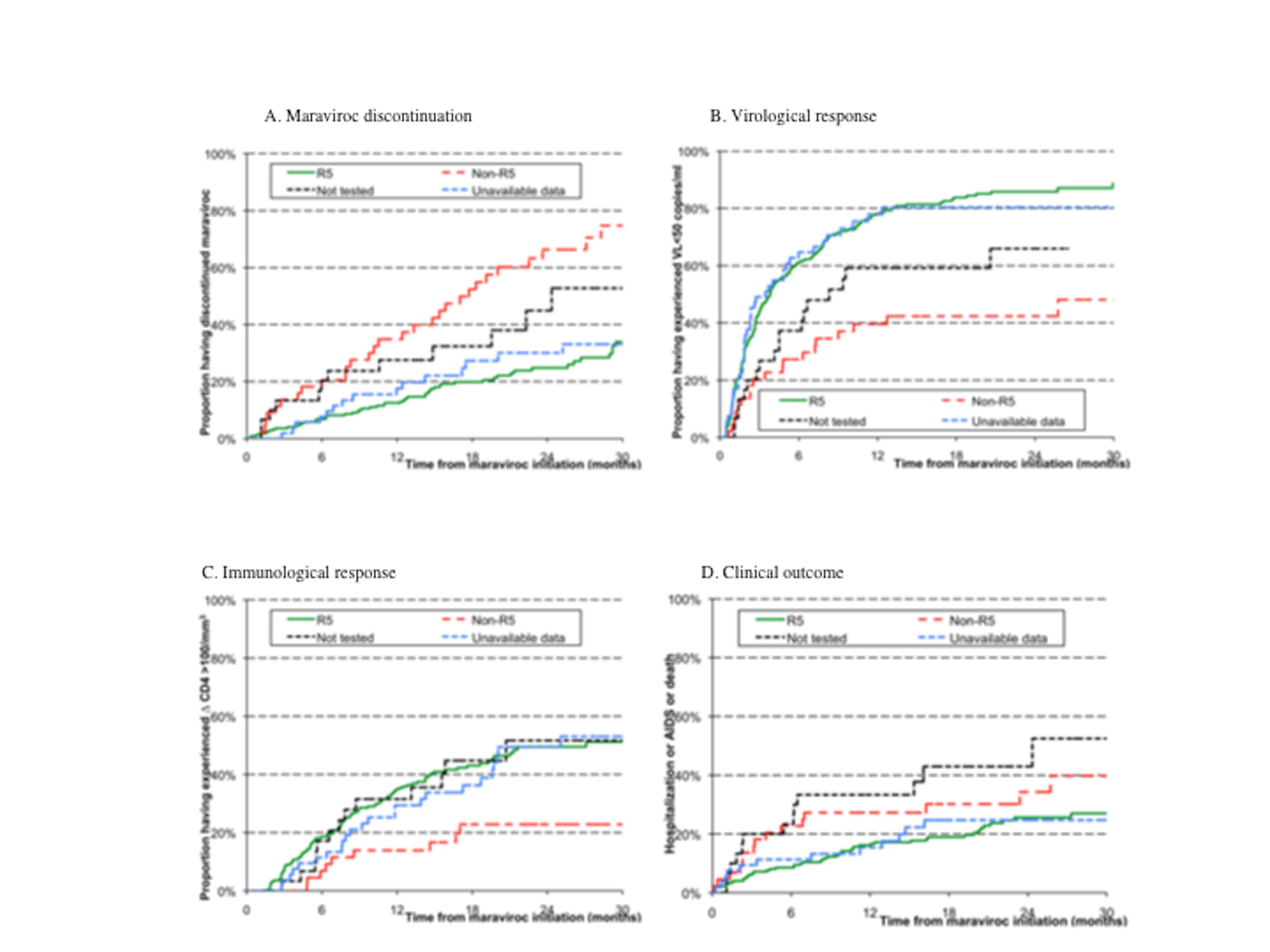

Supplement: S1 File — (TIF) [file pone.0144746.s001.tif]
