# Supplementary material for: Durability and Effectiveness of Maraviroc-Containing Regimens in HIV-1-Infected Individuals with Virological Failure in Routine Clinical Practice
Source: PLoS One. 2015 Dec 29;10(12):e0144746. doi: 10.1371/journal.pone.0144746 (PMC4695083; doi:10.1371/journal.pone.0144746)
Supplement: S1 Text — (PDF) [file pone.0144746.s002.pdf]

Appendix

Clinical Epidemiology Group of the FHDH-ANRS CO4

- **Scientific committee**: S Abgrall, F Barin, E Billaud, F Boué, L Boyer, A Cabié, F Caby, A Canestri, D Costagliola, L Cotte, P De Truchis, X Duval, C Duvivier, P Enel, H Fischer, J Gasnault, C Gaud, J Gilquin, S Grabar, MA Khuong, O Launay, A Mahamat, M Mary-Krause, S Matheron, G Melica-Gregoire, H Melliez, JL Meynard, J Pavie, L Piroth, I Poizot-Martin, C Pradier, J Reynes, E Rouveix, A Simon, P Tattevin, H Tissot-Dupont, JP Viard.
- **COREVIH coordinating center**: French Ministry of Health (G Astier, D Martin, I Prade), Technical Hospitalization Information Agency, ATIH (N Jacquemet).
- **Statistical analysis center**: UMRS 1136 INSERM et UPMC (D Costagliola, *Principal investigator*, S Abgrall, S Grabar, M Guiguet, S Lang, L Lièvre, M Mary-Krause, H Selinger-Leneman), INSERM-Transfert (JM Lacombe, V Potard).
- **COREVIH:** <u>Paris area</u>: ***Corevih Ile de France Centre*** (GH Pitié-Salpétrière: O Benveniste, A Simon, G Breton, C Lupin, E Bourzam; Hôpital Saint-Antoine: PM Girard, L Fonquernie, N Valin, B Lefebvre, M Sebire; Hôpital Tenon: G Pialoux, MG Lebrette, P Thibaut, A Adda, M Hamidi, J Cadranel, A Lavolé, A Parrot), ***Corevih Ile de France Est*** (Hôpital Saint-Louis: E Oksenhendler, L Gerard, JM Molina, W Rozenbaum, B Denis, N De Castro, C Lascoux; GH Lariboisière-Fernand Widal: JF Bergmann, V Delcey, A Lopes, P Sellier, M Parrinello; Hôpital Avicenne: O Bouchaud, N Vignier, F Méchaï, S Makhloufi, P Honoré; Hôpital Jean Verdier), ***Corevih Ile de France Nord*** (Hôpital Bichat-Claude Bernard: Y Yazdanpanah, S Matheron, S Lariven, V Joly, C Rioux; Hôpital Delafontaine: MA Khuong-Josses, M Poupard, B Taverne), ***Corevih Ile de France Ouest*** (Hôpital Ambroise Paré: E Rouveix, S Greffe, C Dupont, A Freire Maresca, E Reimann; Hôpital Louis Mourier: M Bloch, F Meier, E Mortier, F Zeng, B Montoya; Hôpital Raymond Poincaré: C Perronne P de Truchis, D Mathez, D Marigot-Outtandy, H Berthé; CH André Mignot - Le Chesnay: A Greder Belan, A Therby, C Godin Collet, S Marque Juillet, M Ruquet, S Roussin-Bretagne, P Colardelle; Hôpital Foch – Suresnes: JE Kahn, D Zucman, C Majerholc, E Fourn, D Bornarel; Hôpital Victor Dupouy – Argenteuil: L Sutton, V Masse, P Genet, B Wifaq, J Gerbe; Hôpital Max Fourestier – Nanterre: V Daneluzzi, J Gerbe; CHI de Meulan les Mureaux: T Akpan, M Marcou; CH F Quesnay - Mantes La Jolie: F Granier, JJ Laurichesse, V Perronne; CHI de Poissy: C Veyssier-Belot, H Masson; CHI de St-Germain-en-Laye: Y Welker, P Brazille), ***Corevih Ile de France Sud*** (Hôpital Européen Georges Pompidou: L Weiss, J Pavie, MI Lucas, C Jung, M Ptak; GH Tarnier-Cochin: D Salmon, C Le Jeunne, O Launay, P Guet, MP Pietri, E Pannier Metzger, V Marcou, P Loulergue, N Dupin, JP Morini, J Deleuze, P Gerhardt, J Chanal; Hôtel Dieu: JP Viard, J Ghosn, P Gazalet, A Cros, A Maignan; Hôpital Antoine Béclère: F Boué, S Abgrall, V Chambrin, I Kansau, M Raho-Moussa; Hôpital de Bicêtre: D Vittecoq, O Derradji, C Bolliot, C Goujard, E Teicher, J Gasnault, M Mole, K Bourdic; Hôpital Henri Mondor: JD Lelievre, G Melica, M Saidani, C Chesnel, C Dumont; Hôpital Necker: C Duvivier, O Lortholary, C Rouzaud, F Touam, K Benhadj; CMIP Pasteur: PH Consigny, P Bossi, A Gergely, G Cessot, F Durand).

  <u>**Outside Paris area**</u>: ***Corevih Alsace*** (CHU de Strasbourg: D Rey, M Partisani, C Cheneau, ML Batard, P Fischer; CH de Mulhouse: G Beck-Wirth, C Michel, M Benomar), ***Corevih de l'Arc Alpin*** (CHU de Grenoble: P Leclercq, M Blanc, P Morand, O Epaulard, A Signori-Schmuck), ***Corevih Auvergne-Loire*** (CHU de Clermont-Ferrand: H Laurichesse, C Jacomet, M Vidal, D Coban, S Casanova; CHU de Saint-Etienne: A Fresard, C Guglielminotti, E Botelho-Nevers, A Brunon-Gagneux, V Ronat); ***Corevih Basse-Normandie*** (CHU de Caen: R Verdon, S Dargère, E Haustraete, P Féret, P Goubin), ***Corevih Bourgogne*** (CHU de Dijon: P Chavanet, A Fillion, L Piroth, D Croisier, S Gohier), ***Corevih Bretagne*** (CHU de Rennes: C Arvieux, F Souala, JM Chapplain, M Ratajczak, J Rohan), ***Corevih Centre et Poitou Charentes*** (CHRU de Tours), ***Corevih Franche-Comté*** (CHRU de Besançon: C Chirouze, L Hustache-Mathieu, JF Faucher, A Proust, N Magy-Bertrand, H Gil, N Méaux-Ruault; CH de Belfort: JP Faller, O Ruyer, V Gendrin, L Toko); ***Corevih Haute-Normandie*** (CHU de Rouen), ***Corevih Languedoc-Roussillon*** (CHU de Montpellier; CHU de Nîmes: A Sotto, I Rouanet, JM Mauboussin, R Doncesco, G Jacques), ***Corevih Lorraine Champagne-Ardennes*** (Nancy Hôpital de Brabois: T May, C Rabaud, M Andre, M Delestan, MP Bouillon; CHU de Reims), ***Corevih de Midi-Pyrénées Limousin*** (Toulouse CHU Purpan: B Marchou, P Delobel, G Martin Blondel, L Cuzin, N Biezunski, L Alric, D Bonnet, M Guivarch, A Palacin, V Payssan), ***Corevih Nord-Pas de Calais*** (CH de Tourcoing: H Melliez, F Ajana, A Meybeck, N Viget), ***Corevih PACA Est*** (Nice Hôpital Archet 1: C Pradier, P Pugliese, PM Roger, E Rosenthal, J Durant, E Cua, A Naqvi, I Perbost, K Risso; CH Antibes-Juan les Pins: D Quinsat; CHI de Fréjus/St Raphaël: P Del Giudice; CH de Grasse: PY Dides), ***Corevih***

PACA Ouest (Marseille Hôpital de la Conception: P Enel, R Sambuc, MS Antolini-Bouvenot, P Druart, L Meddeb, I Ravaux, A Menard, C Tomei, C Dhiver, H Tissot-Dupont; Marseille Hôpital Nord: J Moreau, S Mokhtari, MJ Soavi, V Thomas; Marseille Hôpital Sainte-Marguerite: I Poizot-Martin, S Bregigeon, O Faucher, V Obry-Roguet, AS Ritleng, N Petit; Marseille Centre pénitentiaire des Baumettes: C Bartoli, JM Ruiz, D Blanc; CH d'Aix-En-Provence: T Allegre, M Sordage, JM Riou, C Faudon; CH d'Avignon: B Slama, H Zerazhi, O Boulat, S Chebrek, M Beyrne; CH de Digne Les Bains: P Granet Brunello; CH de Gap: L Pellissier, D Bonnabel; CH de Martigues: R Cohen Valensi, B Mouchet, G Mboungou; CHI de Toulon: A Lafeuillade, E Hope-Rapp, G Hittinger, G Philip, V Lambry), ***Corevih Pays de la Loire*** (CHU de Nantes: F Raffi, C Allavena, E Billaud, N Hall, V Reliquet), ***Corevih de la Vallée du Rhône*** (Lyon Hôpital de la Croix-Rousse: C Chidiac, L Cotte, T Ferry, T Perpoint, P Miailhes; Lyon Hôpital Edouard Herriot: A Boibieux, JM Livrozet, D Makhloufi, F Brunel, P Chiarello).
Overseas: ***Corevih Guadeloupe*** (CHU de Pointe-à-Pitre: B Hoen, I Lamaury, I Fabre, K Samar, E Duvallon; CH Saint-Martin: C Clavel, S Stegmann, V Walter), ***Corevih Guyane*** (CH de Cayenne: M Nacher, L Adriouch, F Huber, V Vanticlke, P Couppié), ***Corevih Martinique*** (CHU de Fort-de-France: A Cabié, S Abel, S Pierre-François), ***Corevih de La Réunion*** (CHU Félix Guyon: C Gaud, C Ricaud, R Rodet, G Wartel, C Sautron; GH Sud Réunion: P Poubeau, G Borgherini, G Camuset).
